# Outcome of early rehabilitation of patients with traumatic brain injury during COVID-19 pandemic in The Republic of Srpska, Bosnia and Herzegovina

**DOI:** 10.3389/fneur.2023.1269564

**Published:** 2023-09-28

**Authors:** Nataša Keleman, Rastislava Krasnik, Aleksandra Mikov, Dragana Dragičević-Cvjetković

**Affiliations:** ^1^Clinical Rehabilitation Service, University Clinical Center of the Republic of Srpska, Banja Luka, The Republic of Srpska, Bosnia and Herzegovina; ^2^Faculty of Medicine, University of Novi Sad, Novi Sad, Serbia; ^3^Clinic for Children Habilitation and Rehabilitation, Institute for Children and Youth Health Care of Vojvodina, Novi Sad, Serbia; ^4^Department of Medical Rehabilitation and Balneoclimatology, Institute for Physical Medicine, Rehabilitation and Orthopedic Surgery “Dr Miroslav Zotović”, Banja Luka, Bosnia and Herzegovina; ^5^Faculty of Medicine, University of Banja Luka, Banja Luka, The Republic of Srpska, Bosnia and Herzegovina

**Keywords:** traumatic brain injury, COVID-19 pandemic, epidemiology, early rehabilitation, clinical outcome

## Abstract

**Introduction:**

The COVID-19 pandemic has placed a tremendous burden on the healthcare system. Patients with traumatic brain injury (TBI) have to get fast track treatment which is independent of environmental conditions. The aim of this study was to investigate epidemiological and clinical outcomes of early rehabilitation and compare it with the literature data during the non-COVID-19 period.

**Materials and methods:**

A retrospective study included 174 patients with TBI, average 57 ± 19.08 years. They all underwent treatment in the University Clinical Center, Republic of Srpska, Bosnia and Herzegovina during the period January-December 2021. We have analyzed the epidemiological data and clinical course in 174 patients as well as the outcome of early rehabilitation in 107 patients. In clinical evaluation were used: Glasgow Coma Score (GCS), Functional Independence Measure (FIM) and Barthel Index on admission and at discharge, as well as Glasgow Outcome Scale (GOS) at discharge. ANOVA, SPANOVA, Student *t-*test and Pearson correlation coefficient were used in statistical analysis. The value (*p* < 0.05) was used for statistical significance.

**Results:**

A total of 174 patients with TBI were included in this study. Most of the patients (*n* = 94) were older than 60, male (*n* = 125) and the most frequent cause of TBI was falling over (*n* = 88). About a half (*n* = 92) had a mild TBI, almost one third of the sample had moderate (*n* = 52), while only 30 patients had severe TBI. Total of 139 (80.3%) patients had the improved outcome, the worsening was registered in 2 (1.2%), while the fatal outcome was reported with 33 (18.5%) patients. When comparing the scores on admission and at discharge, the improvement of mean parameter values was reported for GCS (9.9 vs. 14.1), for Barthel Index (57.25 vs. 86.85), and for FIM (67.35 vs. 105.15), (*p* < 0.001). A complete recovery at discharge was found in 63.79%, a mild deficit in 8.62%, while serious deficit was found with 6.32%, and vegetative state with 2.29% patients.

**Conclusion:**

The COVID-19 pandemic had a significant effect on the epidemiological data but not on the clinical outcome of patients with TBI. Early rehabilitation proved to be effective and to contribute to positive treatment outcome.

## 1. Introduction

Traumatic brain injury (TBI) is one of the leading causes of morbidity, disability and mortality in all age groups, placing a significant burden on healthcare systems. There are more than 50 million people experiencing a brain injury in the world on the annual basis. The incidence of TBI in Europe is 47.3–694 per 100,000 inhabitants per year, while the mortality rate is 9–28.1/100,000 (1). The direct and indirect costs of TBI treatment in the USA amount to 90 billion dollars, and in the Netherlands 383.81 million Euro (2, 3). TBI is classified according to the mechanism of injury, location and severity of the injury. Regarding TBI severity, the Glasgow Coma Scale (GCS) is most often used on admission. According to this scale, brain injuries are divided into mild (GCS ≥ 13), moderate (GCS 9–12) and severe (GCS < 9). There is currently no specific therapy for TBI. Patients with moderate and severe brain injury require complex treatment and rehabilitation involving a multidisciplinary approach (4). However, even patients with a mild brain injury can develop a severe clinical picture, which is why a prognostic model is necessary to plan their early rehabilitation. Unfortunately, there is still no ideal predictive model that would include clinical outcomes or biomarkers (5).

TBI leads to physical, cognitive, social, emotional and behavioral disorders. This is why in recent years there has been more and more interest in the effectiveness of early rehabilitation in reducing these disorders (6–8). Outcomes after TBI can range from full recovery to permanent disability or death. Even mild TBI can impair neuronal integrity, alter brain metabolism, and increase cell membrane renewal, which can cause long-term neurodegeneration (9).

Early rehabilitation can begin very early, after the patient has been stabilized. Early neurorehabilitation aims to improve motor and functional recovery while preventing secondary complications (pneumonia, atelectasis, muscle atrophy, decubitus ulcers, deep vein thrombosis, contractures). The intensity and duration of early rehabilitation are recommended to be individually dosed depending on the patient's general condition and abilities, and the approach should be multidisciplinary (10).

Measuring rehabilitation treatment outcomes after TBI is particularly challenging due to the variety and severity of impairments that remain after hospitalization and the post-acute period. The following scales are most commonly used: Glasgow Coma Scale (GCS), Glasgow Outcome Scale (GOS), Functional Independence Measurement (FIM) and Barthel Index (11, 12). The COVID-19 pandemic has led to severe health challenges with major socioeconomic consequences around the world. Due to COVID-19 healthcare systems had to adapt quickly to the increased influx of patients, as well as the increased need for respiratory and intensive care. Due to the exponential admission to hospitals and intensive care units, it was necessary to redistribute staff, and to change the usual working practices. Due to the maximum occupancy of the intensive care units (ICU) and the increased need for mechanical ventilation, the operating theaters were converted into improvised intensive care units (13). To date, it remains unclear to what extent the pandemic and the measures implemented have affected the treatment and outcome of patients suffering from brain injury, especially due to the fact that moderate and severe brain injuries require hospitalization in ICU. The first case of COVID-19 infection in the Republic of Srpska was registered on 5 March 2020, vaccination started on 9 March 2021, and a lockdown was introduced on 21 March 2020 (14). The epidemiology of TBI was largely influenced by the changes in daily routine. The results of studies conducted in Austria, Italy, France, Finland and Switzerland have shown a reduced number of TBI cases compared to the years before the pandemic (15–19). According to a recent meta-analysis, underdeveloped and medium-developed countries were the most affected, where mortality due to TBI increased (20). Despite the lockdowns, the number of traffic accidents in some countries was reported to paradoxically increase (21). According to our information, in Bosnia and Herzegovina has been no research on the outcome of early rehabilitation of the patients suffering from TBI to date. Two studies on epidemiological and clinical characteristics of TBI have been published in Bosnia and Herzegovina. One hundred and forty one patients suffering from severe TBI, during the period 2002–2004 were described in the study Dizdarević et al. (22). A multi-center study on TBI, published in 2007, compared the epidemiological characteristics, treatment and outcome of the severe TBI in the European countries with various economic statuses, whereby Bosnia and Herzegovina was among less developed countries (23). The aim of this study was to investigate epidemiological and clinical outcomes of early rehabilitation and compare it with the literature data during non COVID-19 period.

## 2. Materials and methods

**Figure 1:**
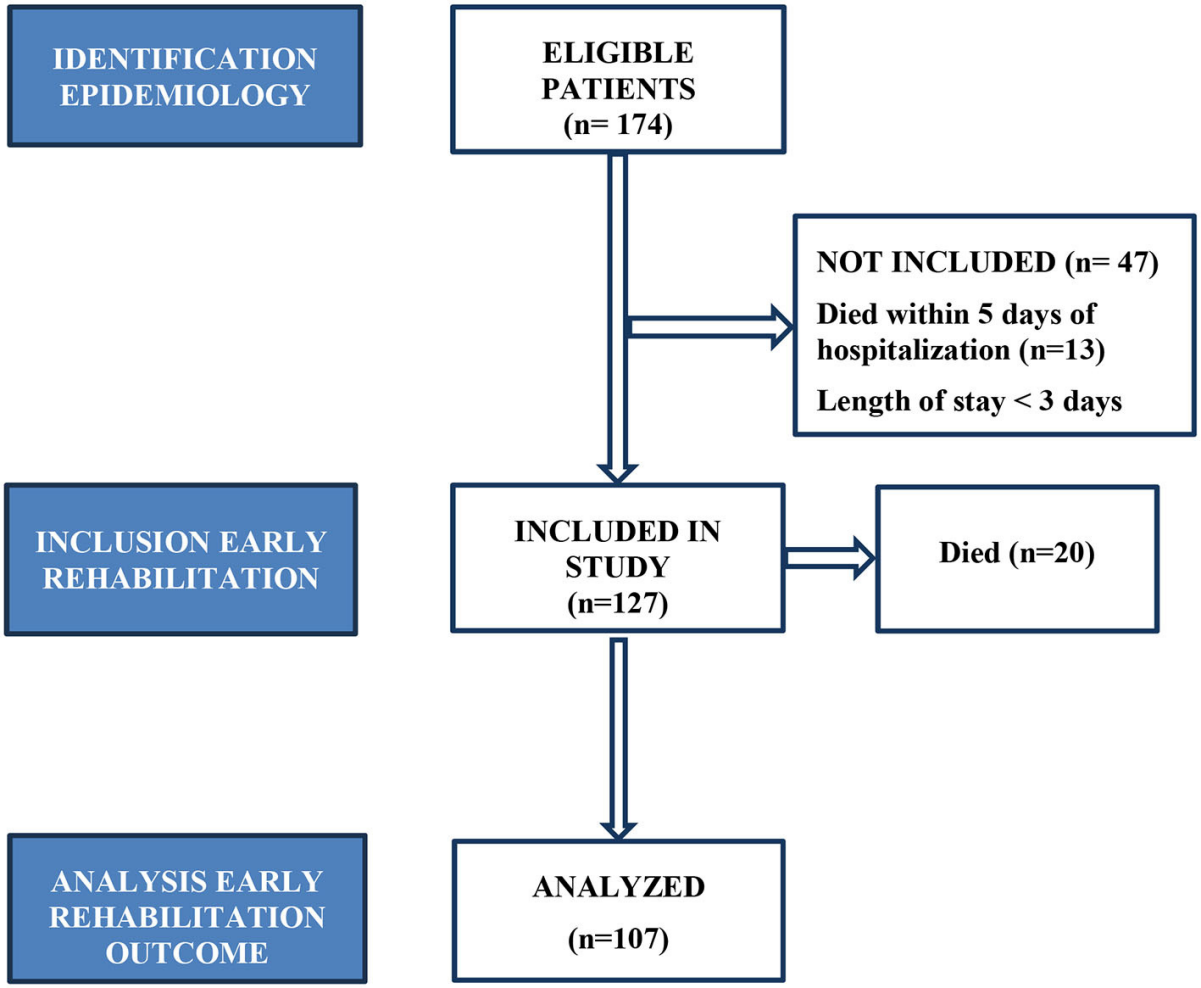
Flow diagram representing study design with inclusion and exclusion criteria.

In our research we conducted a retrospective study that included all patients suffering from TBI admitted to the Intensive Care Unit and Neurosurgery Clinic of the Republic of Srpska University Clinical Center, Banja Luka, Bosnia and Herzegovina during the period from 1 January 2021 to 31 December 2021. About 1,170,000 inhabitants live in the region covered by the Republic of Srpska University Clinical Center, which is the reference institution (24). The inclusion criteria implied the patients suffering from TBI, Covid-19 negative. 174 patients whose epidemiological data and treatment outcome were analyzed were included in the study. The excluding criteria for early rehabilitation were: the patients who died during the first 5 days of being hospitalized (*n* = 13), length of stay < 3 days (*n* = 34) and Covid-19 positive patients (*n* = 0). 20 patients died during early rehabilitation, so that the outcomes of early rehabilitation were analyzed with 107 patients (Figure 1).

We analyzed the following parameters: sex, age, mechanism and type of injury, number of surgical procedures, number of patients on mechanical ventilation, number of total days of hospitalization, outcome and implementation of early rehabilitation. The mentioned variables represent independent research variables. The kinesiotherapy implementation plan included positioning, an anti-decubitus program, passive and actively assisted exercises to maintain and increase the range of motion in all segments of the upper and lower extremities, as well as respiratory kinesiotherapy. Early rehabilitation began after stabilization of the patients, on the second day at the earliest, and on the tenth day after admission at the latest. It was conducted twice a day for 30 min, 7 days a week. Dependent variables relate to the outcome of the treatment measured by the following methods: Glasgow Coma Scale (GCS) on admission and discharge, Glasgow Outcome Scale (GOS) on discharge, Functional Independence Measurement (FIM) on admission and discharge, and Barthel's Index on admission and discharge. The GCS is a point-based pathophysiological score that determines the level of consciousness after a brain injury by assessing verbal and motor responses as well as eye opening. It is expressed in points, with the lowest score being 3, which indicates deep coma or death, and the highest being 15, which indicates that the central nervous system function of a patient has been preserved. Considering the total number of GCS, all brain injuries are divided into mild GCS (≥13), moderate (GCS 9–12) and severe (GCS < 9) (10). The Glasgow Outcome Scale (GOS) is used to assess disability after brain injury. Five outcome categories were distinguished: death, vegetative state, severe deficit, mild deficit and complete recovery (12). The functional independence index measures the motor and cognitive functioning of the patient, and includes 18 parameters for the assessment of physical, psychological and social functions. The parameters are divided into six groups: self-care, sphincter control, mobility, motor skills, communication and social cognition. Each of the 18 items is rated from 1 (complete assistance) to 7 (complete independence). The maximum number of points is 126 (complete independence), and the minimum number of points is 18 (complete dependence). According to the score, the patients are categorized into seven categories: 1. Full assistance, 2. Maximum assistance, 3. Medium assistance, 4. Minimal assistance, 5. Supervision, 6. Partial independence and 7. Complete independence (2). Barthel's Index serves to assess the functional status in the daily life activities. The Barthel Index assesses personal hygiene, bathing, toileting, climbing stairs, dressing, stool and urination control, transfer from chair to bed, mobility and wheelchair mobility. Based on the obtained results, the patients are assessed as completely independent (100), slightly dependent (91–99), moderately dependent (61–90), severely dependent (21–60) and completely dependent (0–20) (25, 26).

## 3. Statistical analysis

The incidence of individual groups, as well as their share in percentages was calculated for all independent research variables: gender, age (years), mechanism of injury, type of injury, surgery, mechanical ventilation, early rehabilitation, total hospitalization days, outcome. All listed independent variables are categorical: The Kolmogorov–Smirnov (K-S) test was used to test the normality of the distribution of numerical variables, while Skewness and Kurtosis were also used as the measures of distribution asymmetry, for the following variables: Barthel index at admission, Barthel index at discharge, FIM at admission, FIM at discharge, GCS at admission, GCS at discharge as well as GOS. The psychometric properties of the tests were examined, whereby Cronbach α was used to examine internal consistency. The value of the test α ≥ 0.7 is considered as the acceptable test reliability. Of descriptive indicators, median (Me) with interquartile range (IQR), arithmetic mean (M) with standard deviation (SD) as well as a range (Minimum - Maximum) were shown.

In order to determine whether there were statistically significant differences in the prominence of dependent variables, while taking into account the independent variables (gender, age categories, mechanism of injury, type of injury, surgical procedure, mechanical ventilation, early rehabilitation, number of hospitalization days and outcome), Student's *t*-test and ANOVA were used. Both tests are two-tailed tests for the possibility of an effect in two directions—positive and negative. Split-plot ANOVA (SPANOVA) was used to investigate the effects of independent variables on repeated measures. In other words, the influence of sex, age, mechanism of injury, type of injury, presence of surgeries, mechanical ventilation and early rehabilitation on the outcome of treatment was examined, measured by the Barthel scale, FIM and GCS. SPANOVA test was also used to test a separate recovery, i.e., the difference at the beginning and at the end of measuring of Barthel test, FIM and GCS. Wilks' λ and its statistical probability (p) were shown for the influence of the interaction of independent and dependent variable (e.g. whether the change of the result on Barthel test is equal for men and women). Partial η^2^ was used to measure the size of the effect of independent variables on the change of the results at the beginning and at the end of measurement for the Barthel test, FIM and GCS. According to Cohen criterion, the influence can be small (Partial η ^2^ = 0.01), medium (Partial η ^2^ = 0.06) and large (Partial η ^2^ = 0.14). Wilks' λ is also an indicator of a separate influence of time, i.e. the difference at the beginning and at the end for the measured parameters. Correlations between numerical variables were tested by the Pearson correlation coefficient and displayed using a Scatter Plot. The level of statistical significance was set at *p* < 0.001. IBM SPSS Statistics version 25 was used for data analysis.

## 4. Results

A total of 174 patients with TBI were included in the research, of which 28.2% were female. A total of 4.6% of these patients were under 20 years of age, 14.9% were 21 to 40 years old, 26.4% of patients were 41 to 60 years old, while more than a half of the patients were over 60 years old (54.0%). Falling over was the cause of half of the TBI, or 50.6%. Subdural hematoma (SDH) was present in 38.5%, contusion in 35.6%, epidural hematoma (EDH) in 14.9%, subarachnoid hemorrhage (SAH) in 8.6%, while 2.3% of patients suffered from intracerebral hematoma (ICH). One third (33.3%) of patients with TBI during the COVID-19 pandemic underwent a surgery, while 20.7% of patients were on mechanical ventilation. According to the GCS, 92 (52.87%) had a mild brain injury, 52 (29.88%) had a moderate brain injury, and 30 (17.25%) had a severe brain injury. Due to the general condition of 127 (73.0%) patients early rehabilitation was possible. Improvement was recorded in 80.3% of patients, symptoms worsened with 1.2%, while death was recorded in 18.5% of patients. Worsening and improvement were defined based on the clinical condition of the patient at discharge. General data of these patients are shown in Table 1. Table 2 shows the differences in functional scores in the examined patient sample in relation to epidemiology, injury mechanism and treatment outcome.

**Table 1:** General data on patients with traumatic brain injury.

	***N* = 174**
**Gender**	
Male	125 (71.8%)
Female	49 (28.2%)
**Age (years)**	
0–20	8 (4.6%)
21–40	26 (14.9%)
41–60	46 (26.4%)
>60	94 (54.0%)
**Mechanism of injury**	
Traffic trauma	23 (13.2%)
Fall	88 (50.6%)
Other cases	55 (31.6%)
No data	8 (4.6%)
**Type of injury**	
Contusion	62 (35.6%)
Epidural haematoma	26 (14.9%)
Subdural haematoma	67 (38.5%)
Subarachnoid hemorrhage	15 (8.6%)
Intracerebral haematoma	4 (2.3%)
**Surgery**	
Yes	58 (33.3%)
No	116 (66.7%)
**Mechanical ventilation**	
Yes	36 (20.7%)
No	138 (79.3%)
**Early rehabilitation**	
Yes	127 (73.0%)
No	47 (27.0%)
**Total days of hospitalization**	
0–3	34 (19.5%)
4–7	57 (32.8%)
8–10	31 (17.8%)
>11	52 (29.9%)
**Outcome**	
Improved	139 (80.3%)
Deceased	33 (18.5%)
Worsening	2 (1.2%)

Worsening and improvement were defined based on the clinical condition of the patient at discharge.

**Table 2:** Differences among the functional scores in patients with traumatic brain injury regarding the epidemiology, mechanism of injury and outcome of treatment.

	**BA**	**BD**	**FIMA**	**FIMD**	**GCSA**	**GCSD**	**GOS**
**Gender** (*p*-value)^a^	0.055	0.087	0.016	0.097	0.014	0.164	0.161
Male	50.9 (32.2)	83.1 (23)	60 (29.1)	101 (25.8)	9.3 (3.4)	13.8 (2.1)	1.6 (1)
Female	63.6 (24)	90.6 (5.4)	74.7 (22.9)	109.3 (10.2)	10.9 (1.3)	14.4 (1)	1.3 (0.6)
**Age (years)** (*p*-value)^b^	0.226	0.765	0.128	0.679	0.001	0.464	0.51
0–20	57 (29.5)	90.3 (4.6)	66.7 (25.3)	113.8 (2)	9.5 (3.3)	14.8 (0.4)	1 (0)
21–40	44.7 (36.4)	83.3 (23.4)	53 (30.4)	100.9 (27)	7.9 (4)	13.7 (2.3)	1.6 (1)
41–60	49.2 (32.3)	82.9 (23.1)	59.2 (29.8)	102 (25.5)	8.8 (3.3)	13.7 (2.1)	1.5 (0.9)
>60	60 (27.3)	86.5 (18.4)	69.8 (26.1)	103.5 (21.3)	10.8 (1.9)	14.2 (1.8)	1.5 (0.9)
**Mechanism of injury** (*p*-value)^b^	0.208	0.864	0.555	0.603	0.292	0.704	0.457
Traffic injury	56.3 (28.8)	89.8 (6.1)	65.8 (26.2)	114.7 (0.5)	9.3 (3.2)	14.8 (0.4)	1 (0)
Fall	59.1 (29.8)	85.6 (20.6)	66.8 (27.2)	102.3 (23.6)	10.1 (3)	14 (2)	1.6 (0.9)
Other causes	46.6 (29.9)	83.1 (22)	59.2 (29.4)	102.3 (24.4)	9.3 (2.6)	13.9 (2)	1.5 (0.9)
No data	40 (46.2)	87.5 (8.7)	54 (41.6)	108.8 (12.5)	7.5 (5.2)	14.3 (1.5)	1.3 (0.5)
**Type of injury** (*p*-value)^b^	0.007	< 0.001^*^	0.009	0.002	< 0.001^*^	0.002	0.032
Contusion	65 (24.3)	91.4 (5.9)	71.7 (24.5)	108.2 (11.6)	10.3 (2.7)	14.3 (1.1)	1.4 (0.7)
EDH	39.1 (35.4)	80.3 (27.5)	49 (29.2)	100.3 (29.8)	7.6 (3.8)	13.7 (2.4)	1.6 (1)
SDH	53.1 (29.6)	84.4 (19.3)	64 (28.1)	101.9 (22.7)	10.3 (2.3)	14 (1.9)	1.6 (0.9)
SAH	66.5 (24.7)	89.2 (11)	77.3 (22.6)	110 (15.8)	10.4 (2.8)	14.5 (1.6)	1.2 (0.6)
ICH	n/a	n/a	n/a	n/a	n/a	n/a	n/a
**Surgery** (*p*-value)^a^	< 0.001^*^	0.003	0.001	0.006	0.002	0.032	0.014
Yes	39.2 (31.8)	78 (26.3)	53.2 (30.4)	95.7 (29.5)	8.6 (3.7)	13.5 (2.5)	1.8 (1.1)
No	64.1 (25.6)	89.8 (13.1)	70.9 (24.5)	108.1 (15.8)	10.4 (2.3)	14.3 (1.4)	1.3 (0.7)
**Mechanical ventilation** (*p*-value)^a^	< 0.001^*^	< 0.001^*^	< 0.001^*^	< 0.001^*^	< 0.001^*^	< 0.001^*^	< 0.001^*^
Yes	14.3 (29.6)	66.4 (35.2)	28.7 (23.8)	80.2 (36.1)	5.9 (4.2)	12.2 (3)	2.4 (1.1)
No	63.5 (22.4)	89.4 (11.2)	72.1 (22.3)	108.6 (14.4)	10.6 (1.8)	14.4 (1.2)	1.3 (0.7)
**Early rehabilitation** (*p*-value)^a^	0.621	0.812	0.689	0.914	0.715	0.994	0.994
Yes	54.1 (30.8)	85.1 (20.3)	64.1 (28.4)	103.2 (23.1)	9.7 (3.1)	14 (1.9)	1.5 (0.9)
No	65 (14.1)	88.5 (5)	56 (22.6)	105 (14.1)	10.5 (0.7)	14 (1.4)	1.5 (0.7)
**Total days of hospitalization** (*p*-value)^b^	< 0.001^*^	< 0.001^*^	< 0.001^*^	< 0.001^*^	< 0.001^*^	< 0.001^*^	< 0.001^*^
0–3	n/a	n/a	n/a	n/a	n/a	n/a	n/a
4–7	65.9 (21.6)	91.4 (5.3)	72.7 (22.4)	110.8 (9.5)	10.8 (1.7)	14.6 (0.8)	1.2 (0.5)
8–10	62.2 (23.7)	90.4 (5.5)	73.9 (23.1)	110.8 (10)	10.4 (2.3)	14.6 (0.8)	1.2 (0.5)
>11	38.2 (35.1)	75.7 (29.6)	49.2 (30.4)	91.1 (31.5)	8.3 (3.9)	13 (2.6)	2 (1.1)
**Outcome** (*p*-value)^b^	0.541	0.001	0.958	< 0.001^*^	0.549	0.002	0,001
Improved	54.1 (30.8)	86 (18.5)	63.9 (28.3)	104.3 (21.4)	9.7 (3.1)	14.1 (1.8)	1.5 (0.8)
Deceased	n/a	n/a	n/a	n/a	n/a	n/a	n/a
Worsening	67.5 (17.7)	40 (56.6)	65 (35.4)	46.5 (40.3)	11 (1.4)	10 (2.8)	3.5 (0.7)

^a^Student's t-test was performed.

^**b**^ANOVA was performed.

EDH, epidural haematoma; SDH, subdural haematoma; SAH, subarachnoid hemorrhage; ICH, intracerebral haematoma. BA, Barthel admission; BD, Barthel discharge; FIMA, Functional Independence Measurement admission; FIMD, Functional Independence Measurement discharge; GCSA, Glasgow Coma Score admission; GCSD, Glasgow Coma Score Discharge; GOS, Glasgow Outcome Scale.

n/a, not applicable. The values were shown as Mean (Std. Deviation).

^*^Means statistically significant.

Descriptive statistics of all measured tests and the psychometric characteristics of the questionnaire, as well as the Kolmogorov-Smirnov test and Cronbach's α coefficient are shown in Table 3. We tested the normality of the distribution using the Kolmogorov-Smirnov test, since it is recommended to use it for large samples (≥50). Statistically-wise, the distribution of all variables deviates significantly from normal. All scales have high or very high reliability (0.718–0.893) Cronbach's α coefficient.

**Table 3:** Descriptive statistics and psychometric characteristics of the questionnaire.

**Total scores**	**Min-Max**	**Me**	**IQR**	**M**	**SD**	**Skewness**	**Kurtosis**	**K-S**	***p***	**α**
BA	0–80	60.00	25.00	54.32	30.59	−1.06	−0.51	0.284	< 0.001^*^	0.732
BD	0–95	92.00	10.00	85.12	20.11	−3.61	12.98	0.315	< 0.001^*^	0.718
FIMA	18–95	75.00	50.00	63.96	28.21	−0.74	−1.08	0.220	< 0.001^*^	0.813
FIMD	18–120	115.00	20.00	103.25	22.90	−2.60	6.83	0.307	< 0.001^*^	0.858
GCSA	3–15	11.00	2.00	9.69	2.96	−2.28	5.18	0.313	< 0.001^*^	0.860
GCSD	7–15	15.00	1.00	13.96	1.87	8.51	83.56	0.452	< 0.001^*^	0.893
GSO	1–4	1.00	1.00	1.50	0.87	1.59	1.38	0.419	< 0.001^*^	0.776

Me, Median; IQR, Inter-quartile range; M, Mean; SD, Std. Deviation; K-S, Kolmogorov–Smirnov test; p, statistical significance; α, Cronbach's alpha; BA, Barthel admission; BD, Barthel discharge; FIMA, Functional Independence Measurement admission; FIMD, Functional Independence Measurement discharge; GCSA, Glasgow Coma Score admission; GCSD, Glasgow Coma Score Discharge; GOS, Glasgow Outcome Scale. ^*^, statistically significant.

An improvement was found at discharge measured by Barthel's index in patients with TBI [Wilks' λ = 0.438, F (1.106) = 135.814, *p* < 0.001, Partial η^2^ = 0.562]. η^2^ accounts for 56.2% of the variance which is classified as a large effect. The influence of early rehabilitation on the value of the test was not recorded, and the same applied to sex, age, mechanism of injury and type of injury. The presence of surgery shows a statistically significant influence on the change in the value of Barthel's Index [Wilks' λ = 0.945, F (1.105) = 6.161, *p* = 0.015, Partial η^2^ = 0.055], as well as mechanical ventilation [Wilks' λ = 0.858, F (1.105) = 17.328, *p* < 0.001, Partial η^2^ = 0.142]. The size of the effect of surgery is medium (5.5% variance), and of mechanical ventilation high (14.2% variance). Patients who did not have surgery and who were not on mechanical ventilation had a higher score on the Barthel Index at discharge. The FIM is better at discharge M = 103.25 (22.90) than at admission [M = 63.96 (28.21), Wilks' λ = 0.280, F (1.106) = 271.955, *p* < 0.001, Partial η^2^ = 0.720]. The size of effect of FIM is high (72.0% of variance). The value of the FIM test is influenced by mechanical ventilation [Wilks' λ = 0.943, F (1.105) = 6.398, *p* = 0.013, Partial η^2^ = 0.057]. Mechanical ventilation has a medium effect size on FIM, i.e. it accounted for 5.7% FIM based on mechanical ventilation. Recovery measured by the FIM test is lower in patients who were on mechanical ventilation.

A total of 30 patients (17.24%) had a severe brain injury measured by the GCS. The outcome measured by the GCS is influenced by: gender [Wilks' λ = 0.958, F (1.105) = 4.577, *p* = 0.035, Partial η^2^ = 0.042], age [Wilks' λ = 0.807, F (3.103) = 8.200, *p* < 0.001, Partial η^2^ = 0.193], mechanism of injury [Wilks' λ = 0.916, F (3.103) = 3.163, *p* = 0.028, Partial η^2^ = 0.084], type of injury [Wilks' λ = 0.834, F (4.102) = 5.087, *p* = 0.001, Partial η^2^ = 0.166], surgery [Wilks' λ = 0.952, F (1.105) = 5.240, *p* = 0.024, Partial η^2^ = 0.048] and mechanical ventilation [Wilks' λ = 0.822, F (1.105) = 22.740, *p* < 0.001, Partial η^2^ = 0.178]. Age, mechanical ventilation, type of injury and mechanism of injury have a high impact on GCS, accounting for 19.3% of the variance, 17.8% of the variance, 16.6% of the variance and 8.4% of the variance of the dependent variable, in that sequence. Surgery, accounting for 4.8% of the variance and gender accounting for 4.2% of the variance of the dependent variable, have medium-sized impact on GCS. Better progress measured by measuring by GCS showed better progress in women, the youngest (0–20 years), then in patients whose injuries were caused by traffic trauma, then patients with SAH, and those who did not have surgery and were not on mechanical ventilation. As for the level of consciousness, the results on the GCS showed an improvement in the total sample at discharge compared to admission [Wilks' λ = 0.217, F (1.106) = 382.336, *p* < 0.001, Partial η^2^ = 0.562]. The results are shown in Table 4.

**Table 4:** Outcomes of early rehabilitation of patients with traumatic brain injury.

	**Wilks' Lambda**	***F***	**Hypothesis df**	**Error df**	***p***	**Partial Eta^2^**
Barthel index	0.438	135.814	1	106	< 0.001^*^	0.562
Barthel index × gender	0.993	0.779	1	105	0.379	0.007
Barthel index × age	0.971	1.015	3	103	0.389	0.029
Barthel index × mechanism of injury	0.957	1.547	3	103	0.207	0.043
Barthel index × type of injury	0.944	1.510	4	102	0.205	0.056
Barthel index × surgery	0.945	6.161	1	105	0.015	0.055
Barthel index × mechanical ventilation	0.858	17.328	1	105	< 0.001^*^	0.142
Barthel index × early rehabilitation	0.999	0.144	1	105	0.705	0.001
FIM	0.280	271.955	1	106	< 0.001^*^	0.720
FIM × gender	0.986	1.455	1	105	0.230	0.014
FIM × age	0.943	2.061	3	103	0.110	0.057
FIM × mechanism of injury	0.955	1.610	3	103	0.192	0.045
FIM × type of injury	0.917	2.309	4	102	0.063	0.083
FIM × surgery	0.988	1.229	1	105	0.270	0.012
FIM × mechanical ventilation	0.943	6.398	1	105	0.013	0.057
FIM × early rehabilitation	0.997	0.314	1	105	0.576	0.003
GCS	0.217	382.336	1	106	< 0.001^*^	0.783
GCS × gender	0.958	4.577	1	105	0.035	0.042
GCS × age	0.807	8.200	3	103	< 0.001^*^	0.193
GCS × mechanism of injury	0.916	3.163	3	103	0.028	0.084
GCS × type of injury	0.834	5.087	4	102	0.001	0.166
GCS × surgery	0.952	5.240	1	105	0.024	0.048
GCS × mechanical ventilation	0.822	22.740	1	105	< 0.001^*^	0.178
GCS × early rehabilitation	0.998	0.236	1	105	0.628	0.002

F, F-test; p, statistical significance; FIM, Functional Independence Measurement; GCS, Glasgow Coma Score. ^*^Means statistically significant.

**Figure 2:**
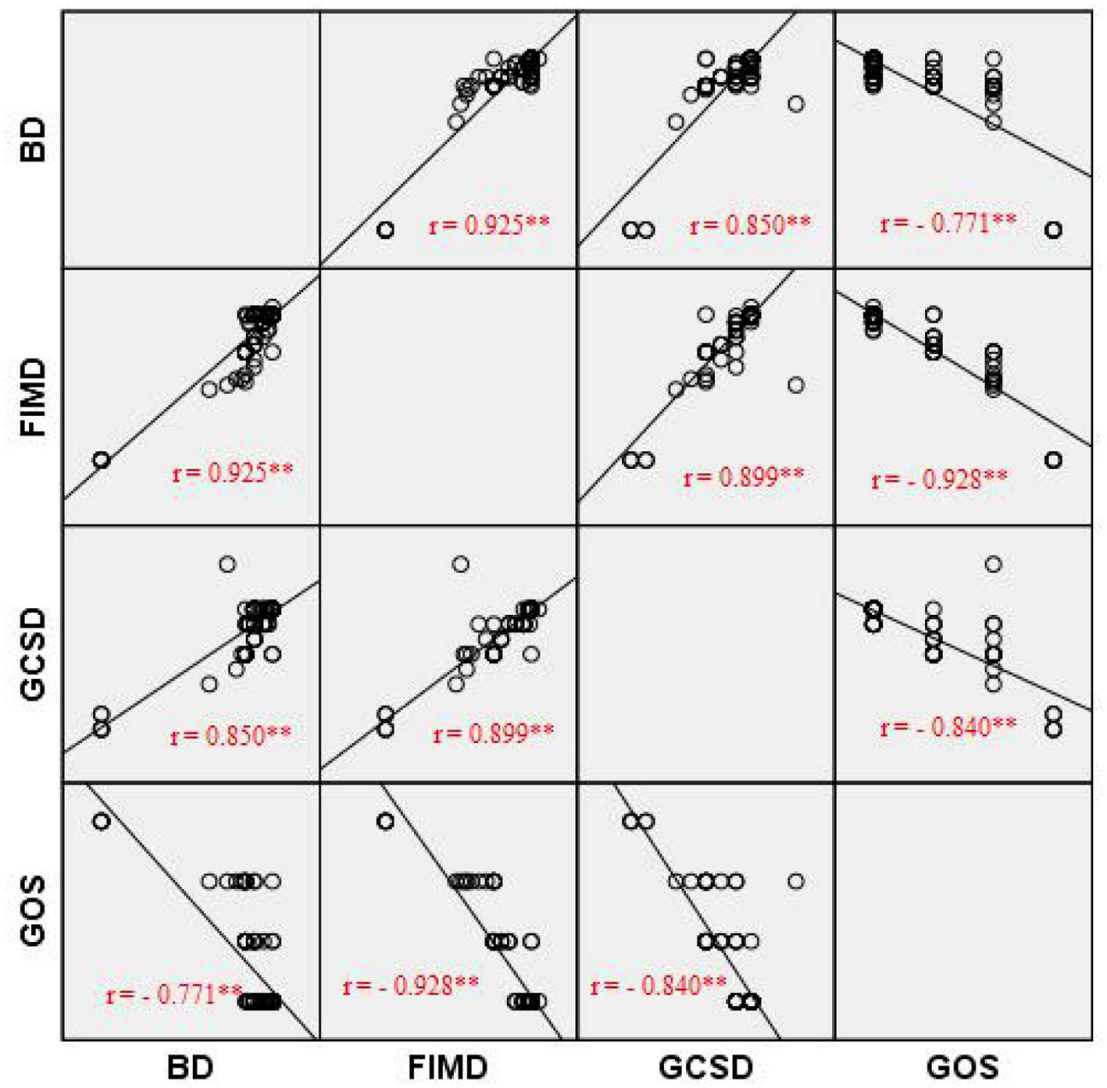
Graph representing correlation between GCS, GOS and Barthel Index. BA, Barthel admission; BD, Barthel discharge; FIMA, Functional Independence Measurement admission; FIMD, Functional Independence Measurement discharge; GCSA, Glasgow Coma Score admission; GCSD, Glasgow Coma Score Discharge; GOS, Glasgow Outcome Scale. Pearson Correlation was performed. **Correlation is significant at the 0.01 level (2-tailed).

Figure 2 shows the relationship between the outcomes studied (BD, FIMD, GCSD and GOS). All correlations are high. GOS negatively correlates with BD (*r* = −0.771, *p* ≤ 0.01), FIMD (*r* = –0.928, *p* ≤ 0.01), GCSD (*r* = –0.849, *p* ≤ 0.01). All correlations between BD, FIMD and GCSD are very high, positive and statistically significant at the *p* ≤ 0.01 level.

## 5. Discussion

The importance of the TBI problem is underestimated due to the lack of research and quality data. We conducted a retrospective study of the patients who sustained TBI and were treated at the Republic of Srpska University Clinical Center, Banja Luka, the largest tertiary center in the Republic of Srpska.

In our study, we determined with statistical significance that TBI had been more common in men 125 (71.8%). The mean age of the respondents was 57 ± 19.08. According to the age structure, TBI was most prevalent in the group of patients over 60 years of age, 94 (54%). The mean age was 33.4 ± 8.9 in the study by Dizdarević et al. and 29 years in a multi-center clinical research (22, 23). The age distribution of TBI in various studies showed that moderate and severe injuries mainly affected young men, most often due to a traffic trauma (27). Falling over is the most common cause of TBI, in 88 (50.6%) patients, and traffic trauma in 23 (13.2%) of patients. Based on the studies conducted for Bosnia and Herzegovina, the most common cause of TBI included traffic trauma and falls (53 vs. 28) (22). According to the multi-center study, violence (both blunt and penetrating) was considerably a more frequent cause of TBI in the low middle-income economies (12%) (23). Possible causes of falls as the leading mechanism of injury in our study included the following: social isolation, anxiety, alcoholism, lock-down, reduced use of public transport. Falling over by the elderly people can also be explained by lack of balance and coordination, poorer vision, the presence of comorbidities, as well as lower availability of the health system in terms of postponing check-ups in all areas.

Changes in the mechanism of TBI during the pandemic differ among countries and are most likely to reflect their socioeconomic status. For example, in Austria during the lockdown there were no TBI reported caused by skiing; at the same time an increased frequency of TBI due to traffic accidents was registered in New York (15, 21). In Italy, no differences in the TBI mechanism were found compared to the period before the pandemic (16). Despite restrictive measures to control the COVID-19 pandemic, the incidence of TBI remained high during the second wave of the pandemic.

In our research, it was determined that subdural hemorrhage was the most common injury, occurring in 67 (38.5%) patients, followed by brain contusion 62 (35.6%). Surgical intervention (craniotomy) was performed in 58 (33.3%) patients, which is less compared to the results of other studies (37–67%) (28). 36 (20.7%) patients required mechanical ventilation. According to the results of this study, surgery and mechanical ventilation are associated with a worse outcome, which is consistent with the results of other studies (29, 30). The average length of hospitalization was 10.86 days (1–61 days). The outcome of TBI was affected by the pandemic in a number of countries. Published studies most frequently investigated the incidence and severity of TBI, but only few studies have shown the impact of the COVID-19 pandemic on the outcome of TBI (31). Therefore, we can only partially compare the results of our study with any other research.

The incidence of moderate or severe TBI before and during the COVID-19 pandemic varies among studies. A large multi-center study by Grassner et al. concluded that the number of patients requiring neurosurgical intervention was lower during the pandemic compared to previous years (19). A recent meta-analysis found no significant differences in frequency of moderate and severe TBI before and during the pandemic (20).

With 139 (80.3%) patients the outcome was improved, deterioration occurred with 2 (1.2%) patients, while 33 (18.5%) patients died. Reported death rates from TBI vary widely. In the USA, mortality due to TBI was 17.1 per 100,000 people in 2010, while in China, mortality after TBI in 2013 was 13.0 per 100,000 people. Based on Eurostat data from 25 European countries, the mortality rate in 2012 was 11.7/100,000 people (95% CI 9.9–13.6), however ranging widely from 3.6 per 100,000 people in Turkey to 21.8 per 100,000 people in Switzerland (32). In the pre-pandemic period, mortality caused by TBI was 9–28.1/100,000 (12). In a multicentre study conducted in Bosnia and Herzegovina, the mortality from severe TBI was lower than expected: mortality in the ICU was 46%, and according to another study, mortality from severe TBI was 50% to 76.9%, depending on the mechanism of injury in the study by Dizdarević et al. (22, 23).

The mean value of GCS at admission was 9.9, and at discharge 14.1. According to the results of the multi-center study, the mean value of GCS at discharge from ICU was 9.6 (23). In the study conducted by Fan et al. higher GCS was measured in patients who underwent an early intensive rehabilitation program compared to the control group (33). A study by Hankemeier et al. showed that the GCS at discharge in the group of subjects undergoing early rehabilitation was significantly higher compared to the group that did not go through early rehabilitation (25). The mean value of the Barthel Index on admission was 57.25, and at discharge 86.85. In a study that included 623 patients, Hankemeier, et al. showed that a poor outcome was correlated with a Barthel Index of < 50 (25). The average value of the FIM at admission was 67.35, and at discharge 105.15. The study by MacDonald et al. which included 5.582 patients with TBI in the period 2011–2016 showed an increase in FIM values during hospitalization and early rehabilitation of patients (34). Complete recovery at discharge measured by the GOS was reported for 111 (63.79%) patients, mild deficit for 15 (8.62%), severe deficit for 11 (6.32%), vegetative state for 4 (2.29) and death for 33 (18.5%) patients. Steiner et al. found that early rehabilitation was associated with better recovery, based on the GOS (35).

The main advantage of our study is in the size of the sample. The analyzed data were from the relatively long period of COVID-19, which included lockdown and restrictions imposed on movement and social life. Compared to previous studies, we paid special attention to the impact of COVID-19 on the outcome of TBI treatment.

Nevertheless, this study also had some limitations. We conducted a retrospective study in one center. Our center covers approximately 30% of the population with mild TBI and about 90% with moderate and severe TBI. Therefore, we did not fully extend our results to the general population. Most cases of mild TBI were treated in other hospitals. On the other hand, all moderate and severe TBI were referred to our institution. We did not compare the results of early TBI rehabilitation with studies conducted in Bosnia and Herzegovina, because according to our knowledge, such studies have not yet been implemented.

## 6. Conclusion

COVID-19 has imposed many challenges for healthcare professionals in terms of treating TBI patients during the pandemic. According to the results of our study, the COVID-19 pandemic had a significant effect on the epidemiological data rather than on clinical outcome in patients with TBI, because throughout the pandemics, the patients with TBI continued being treated as the highest priority. Early rehabilitation proved to be effective and to contribute to the positive treatment outcome.

## Data availability statement

The raw data supporting the conclusions of this article will be made available by the authors, without undue reservation.

## Ethics statement

The studies involving humans were approved by Ethical Board of University Clinical Center of The Republic of Srpska, Banja Luka, Bosnia and Herzegovina. The studies were conducted in accordance with the local legislation and institutional requirements. Written informed consent for participation was not required from the participants or the participants' legal guardians/next of kin in accordance with the national legislation and institutional requirements.

## Author contributions

NK: Conceptualization, Investigation, Methodology, Writing—original draft. RK: Conceptualization, Methodology, Supervision, Writing—review and editing. AM: Conceptualization, Methodology, Supervision, Writing—review and editing. DD-C: Methodology, Investigation, Supervision, Writing—review and editing.
